# Construction and validation of a prediction nomogram model for acute gastrointestinal failure in patients with severe traumatic brain injury

**DOI:** 10.1097/MD.0000000000041423

**Published:** 2025-02-07

**Authors:** Yadi Mao, Fei Li, Lidi Shen, Chunmin Huang

**Affiliations:** a Department of Neurosurgery, Shaoxing People’s Hospital, Zhongxinbei Road 568th, Yuecheng District, Shaoxing, Zhejiang, China.

**Keywords:** acute gastrointestinal failure, gastrointestinal care, nomogram, severe traumatic brain injury

## Abstract

This study aims to establish and validate the prediction model of acute gastrointestinal failure (AGF) in patients with severe traumatic brain injury. A total of 665 inpatients from Shaoxing People’s Hospital from January 2018 to January 2024 were admitted and randomly divided into training group (466 cases) and validation group (199 cases). Data were collected by general situation questionnaire and AGF assessment tool. According to the results of multivariate logistic regression analysis, the prediction nomogram model was established with R software. Bootstrap method was used for internal verification of the model, and verification group was used for external verification. The area under receiver operating characteristic (ROC) curve, Hosmer–Lemeshow test and calibration curves were used to evaluate the differentiation and calibration degree of the model. Multivariate Logistic regression analysis showed that pulmonary infection, hypoxemia, glasgow coma scale (GCS) score ≤ 8 on admission, hyponatremia and metabolic acidosis were independent risk factors for AGF in patients with severe traumatic brain injury (*P* < .05). On this basis, a new prediction model was constructed, as follows: logit *P* = −4.998 + 0.858 × pulmonary infection + 0.923 × hypoxemia + 1.488 × GCS score ≤ 8 + 1.274 × hyponatremia + 1.020 × metabolic acidosis. The area under ROC of the new model was 0.787 (95% CI: 0.831–0.909), and the cutoff point was 0.4589. The sensitivity and specificity of the model were 69.74% and 76.15%, respectively. Hosmer–Lemeshow goodness of fit test showed that the prediction model had a good fitting effect (*χ* ^2^ = 4.828, *P* = .681). External verification showed that the Hosmer–Lemeshow goodness of fit test showed that the prediction model had a good fitting effect (*χ* ^2^ = 12.712, *P* = .122). Calibration curves showed the nomogram established fits well with the real data. The prediction model constructed in this study has good differentiation and calibration degree, which can intuitively and easily select high-risk patients, and provide reference for early screening and gastrointestinal nursing intervention.

## 1. Introduction

Severe traumatic brain injury (sTBI) is a common critical condition in neurosurgery, characterized by high mortality and disability rates.^[1,2]^ At present, maintenance therapy is the main protocol for this disease, but it is often complicated with gastrointestinal injury of different degrees during treatment.^[3,4]^ Acute gastrointestinal failure (AGF) can be defined as Class III or Class IV acute gastrointestinal injury, which refers to the inability of the body to obtain the necessary fluids and nutrients through the gastrointestinal tract.^[5]^ Its mechanism is that the body is in a state of stress after injury, the sympathetic nerve is excited, the gastrointestinal blood vessels are constricted or even spasm, and the gastrointestinal mucosa is ischemia, which leads to the bleeding and necrosis of the gastrointestinal mucosa. Because these patients cannot eat, need to rely on enteral nutrition, so often appear diarrhea, stomach retention and other different degrees of gastrointestinal complications.^[6,7]^ It has been reported that the incidence of AGF in patients with severe traumatic brain injury is about 30.98%,^[8]^ which can aggravate traumatic brain injury. AGF could lead to malnutrition and prolonged hospital stay, and even lead to multiple organ dysfunction, which is life-threatening. Therefore, early identification of AGF in patients with severe traumatic brain injury is of great significance. At present, clinical attention to such patients complicated with AGF is often insufficient, which is easily ignored by clinical medical staff. Therefore, this study aims to establish a nomogram prediction model by exploring the risk factors for AGF in patients with severe traumatic brain injury, so as to provide reference for clinical prediction, diagnosis and prevention.

## 2. Data and methods

### 2.1. Research object

This study was a retrospective study. Convenience sampling method was used to select patients hospitalized in the Department of Neurosurgery of Shaoxing People’s Hospital from January 2018 to January 2024. Inclusion criteria: meeting the diagnostic criteria of severe traumatic brain injury; age ≥ 18 years old; patients with no history of gastrointestinal ulcer, gastrointestinal bleeding, gastrointestinal tumor, or other clotting disorders. Exclusion criteria: patients with severe heart, lung, liver, and renal insufficiency/failure; severe parenteral injury; patients with severe stress. The sample size was calculated using the logistic independent variable event number method, that is, at least 10 positive cases were required for each predictor included in the final model. This study is expected to include 8 factors, and at least 80 patients are required. According to the incidence of AGF in patients with severe traumatic brain injury is 30.98%, the minimum sample size is 258 cases. Finally, a total of 665 patients who met the inclusion and exclusion criteria were included in this study, and the patients were randomly divided into the training group and the verification group according to a ratio of 7:3, that is, 466 patients in the training group and 199 patients in the verification group.

## 3. Research tools

### 3.1. Questionnaire

3.1.1. The general status questionnaire was designed by ourselves, including age, sex, previous history (hypertension, diabetes), bad living habits (smoking, drinking), injury site, injury cause, infection history (lung, intracranial), GCS score at admission, glucocorticoid use, etc.

3.1.2. Laboratory examination of relevant indicators blood routine, blood biochemistry, blood gas analysis results (hypoxemia, metabolic acidosis), etc.

2.1.3 Glasgow coma scale (GCS)^[9]^ was used to evaluate the neurological function of the patients. The scale includes language response (1–5 points), motor response (1–6 points), and eye opening response (1–4 points), with a total score of 3 to 15 points. The higher the score, the better the recovery of neural function. The GCS score of 7 to 8 is classified as mild coma, 5 to 6 as moderate coma, and 3 to 4 as severe coma. The Cronbach’sα coefficient of GCS is 0.95, and the retest reliability is 0.94, which has good reliability and validity.

### 3.2. Assessment tools

Assessment of hyponatremia.^[10]^ The nursing staff asked the patient to maintain an empty stomach on the test day and take 5 mL of venous blood in the morning to determine the blood sodium level. The serum sodium level was < 135 mmol/L and the duration was > 24 hours. The occurrence of hyponatremia can also be preliminally assessed according to the clinical symptoms of the patient. When the patient has apathy, lethargy, vision, auditory hallucinations or large fluctuations in vital signs, timely examination should be conducted to determine the blood sodium concentration and test once every 24 hours.

## 4. Assessment criteria for acute gastrointestinal failure

### 4.1. Diagnosis and grading criteria of acute gastrointestinal injury

Gastrointestinal dysfunction caused by acute diseases in critically ill patients. ^[11]^ Grade I (risk of gastrointestinal insufficiency or failure) refers to partial impairment of gastrointestinal function, which is characterized by transient symptoms of gastrointestinal dysfunction of clear etiology. Grade II (gastrointestinal insufficiency), gastrointestinal digestion, absorption function cannot meet the body’s needs for nutrients and water, but has not affected the patient’s systemic situation. Grade III (gastrointestinal failure), loss of gastrointestinal function, which cannot be restored and general condition does not improve despite therapeutic intervention. Grade IV (gastrointestinal failure and serious impact on the function of other organs), the development of acute gastrointestinal injury directly endangers the patient’s life, and is accompanied by multiple organ dysfunction and/or shock.

### 4.2 Diagnostic and grading criteria for AGF

AGF is Grade III or IV acute gastrointestinal injury. ^[12,13]^ Mild: intolerance to drinks and food, stomach retention, intestinal sounds weakened or even disappeared; Moderate: obvious flatulence, constipation, positive stool occult blood test; Severe:stress ulcer bleeding or perforation.

## 5. Data collection methods

After this study passed the ethical review of the hospital and obtained the consent of the head of the department, the researchers collected data in the department and patients meeting inclusion and exclusion criteria were screened for inclusion. Clinical data of patients were collected through the hospital medical record system, and all data were cross-reviewed by 2 people every week.

## 6. Statistical methods

SPSS 26.0 (Chicago) and R 4.2.0 were used for statistical analysis. The measurement data were expressed as $\bar{x}\pm s$. One-way analysis of variance was used for comparison among multiple groups. The homogeneity of variance was tested first, and LSD was selected for pound-wise comparison when variance was homogeneity. Measurements of non-normal distributions were represented by the median (M), the first and third quartiles (P25, P75), and the Wilcoxon rank sum test was used for inter-group comparisons. Counting data were expressed as frequency and percentage, and χ^2^ test or Fisher exact test were used for comparison between groups. First, univariate logistic regression analysis is performed, and then the significant factors in the univariate logistic regression analysis are analyzed by multivariate logistic regression analysis. Then the risk factors are introduced into R 4.2.0 software and rms package to construct a nomogram prediction model. Receiver operating characteristic curve was drawn to evaluate the differentiation of this nomogram. Calibration curves were drawn and Hosmer–Lemeshow goodness of fit tests were performed to evaluate the accuracy of this nomogram. *P* < .05 was considered to be statistically significant.

## 7. Result

### 7.1. 1. General features

665 patients with severe traumatic brain injury were divided into training group (466 cases) and validation group (199 cases). In the training group, there were 340 males and 126 females, ranging in age from 39 to 85 years with a median age of 76 years. In the verification group, there were 135 males and 64 females, ranging in age from 28 to 81 years, with a median age of 64 years. Other general information is given in Table 1.

**Table 1:** Univariate logistic regression analysis.

Characteristics	Acute gastrointestinal failure		χ^2^	*P*-value
	No (n = 390)			
	Yes (n = 76)			
Age (yr)				
≥70	129 (33.1)	23 (30.3)	0.229	.632
<70	261 (66.9)	53 (69.7)		
Gender				
Male	256 (65.6)	58 (76.3)	3.298	.069
Female	134 (34.4)	18 (23.7)		
Smoking history				
Yes	88 (22.6)	21 (27.6)	0.911	.34
No	302 (77.4)	55 (72.4)		
Drinking history				
Yes	109 (27.9)	18 (23.7)	0.583	.445
No	281 (72.1)	58 (76.3)		
Hypertension				
Yes	137 (35.1)	26 (34.2)	0.024	.878
No	253 (64.9)	50 (65.8)		
Diabetes				
Yes	49 (12.6)	11 (14.5)	0.207	.649
No	341 (87.4)	65 (85.5)		
Injured area				
Frontal lobe	242 (62.1)	51 (67.1)	3.151	.369
Temporal lobe	88 (22.6)	12 (15.8)		
Parietal lobe	22 (5.6)	7 (9.2)		
other	38 (9.7)	6 (7.9)		
Intracranial infection				
Yes	89 (22.8)	23 (30.3)	1.93	.165
No	301 (77.2)	53 (69.7)		
Pulmonary infection				
Yes	276 (70.8)	64 (84.2)	5.825	.016
No	114 (29.2)	12 (15.8)		
Hypoxemia				
Yes	184 (47.2)	55 (72.4)	16.153	<.001
No	206 (52.8)	21 (27.6)		
Use glucocorticoids				
Yes	49 (12.6)	11 (14.5)	0.207	.649
No	341 (87.4)	65 (85.5)		
GCS score was ≤ 8 on admission				
Yes	198 (50.8)	62 (81.6)	16.153	<.001
No	192 (49.2)	14 (18.4)		
Hyperthermia				
Yes	112 (28.7)	41 (53.9)	18.359	<.001
No	278 (71.3)	35 (46.1)		
Elevated blood pressure				
Yes	144 (36.9)	36 (47.4)	2.927	.087
No	246 (63.1)	40 (52.6)		
Hyperglycemia				
Yes	238 (61.0)	34 (44.7)	6.945	.008
No	152 (39.0)	42 (55.3)		
Hypoproteinemia				
Yes	177 (45.4)	57 (75.0)	22.315	<.001
No	213 (54.6)	19 (25.0)		
Hyponatremia				
Yes	83 (21.3)	36 (47.4)	22.762	<.001
No	307 (78.7)	40 (52.6)		
Cause of injury				
Car accident	174 (44.6)	45 (59.2)	6.375	.095
Fall from high place	51 (13.1)	10 (13.2)		
Tumble	145 (37.2)	18 (23.7)		
Other	20 (5.1)	3 (3.9)		
Hypokalemia				
Yes	67 (17.2)	15 (19.7)	0.287	.592
No	323 (82.8)	61 (80.3)		
Metabolic acidosis				
Yes	227 (58.2)	59 (77.6)	10.125	.001
No	163 (41.8)	17 (22.4)		

Abbreviation: GCS = glasgow coma scale.

## 8. Univariate analysis of acute gastrointestinal failure in patients with severe traumatic brain injury

In this study, patients in the training group were divided into 2 groups for univariate logistic regression analysis based on whether AGF occurred. The results were shown in Table 1. There were no statistically significant differences in age, gender, smoking history, drinking history, hypertension history, diabetes history, injury site, injury cause, intracranial infection, hypertension, and hypokalemia between the 2 groups (*P* > .05); There were statistically significant differences in pulmonary infection, hypoxemia, GCS score ≤ 8 on admission, hyponatremia, metabolic acidosis, glucocorticoid use, elevated body temperature, elevated blood glucose, and hypoproteinemia between the 2 groups (*P* < .05).

## 9. Independent variable assignment

According to the results of the above univariate logistic regression analysis, the factor *P* < .05 in Table 1 was set as the independent variable, and whether the patient had AGF was set as the dependent variable, and the assignment was shown in Table 2.

**Table 2:** Description of variable assignment.

Factor	Variable	Assignment description
Whether acute gastrointestinal failure occurs	Y	Yes = 1; No = 0
Pulmonary infection	X1	Yes = 1; No = 0
Use glucocorticoids	X1	Yes = 1; No = 0
GCS score was ≤ 8 on admission	X2	Yes = 1; No = 0
Hyperthermia	X3	Yes = 1; No = 0
Hyperglycemia	X4	Yes = 1; No = 0
Hypoxemia	X5	Yes = 1; No = 0
Hypoproteinemia	X6	Yes = 1; No = 0
Hyponatremia	X7	Yes = 1; No = 0
Metabolic acidosis	X8	Yes = 1; No = 0

Abbreviation: GCS = glasgow coma scale.

## 10. Multivariate logistic regression analysis of acute gastrointestinal failure in severe traumatic brain injury patient

The results of multivariate logistic regression analysis in Table 3 showed that pulmonary infection, hypoxemia, GCS score ≤ 8 on admission, hyponatremia, and metabolic acidosis were independent risk factors for AGF in patients with severe traumatic brain injury.

**Table 3:** Multivariate logistic regression analysis.

Factor	Β value	Standard error	Wald x^2^	*P* value	OR value	95% CI
Pulmonary infection	0.858	0.359	5.708	.017	2.358	1.167 to 4.766
Hypoxemia	0.923	0.297	9.642	.002	2.517	1.406 to 4.507
GCS score was ≤ 8 on admission	1.488	0.328	20.636	.000	4.429	2.331 to 8.417
Hyponatremia	1.274	0.286	19.837	.000	3.576	2.041 to 6.266
Metabolic cidosis	1.020	0.321	10.129	.001	2.774	1.480 to 5.199
Constant	-4.998	0.562	79.002	.000	0.008	-

Abbreviations: CI = confidence interval, GCS = glasgow coma scale, OR = odds ratio.

## 11. Construction of prediction nomogram model for acute gastrointestinal failure in patients with severe traumatic brain injury

**Figure 1.**
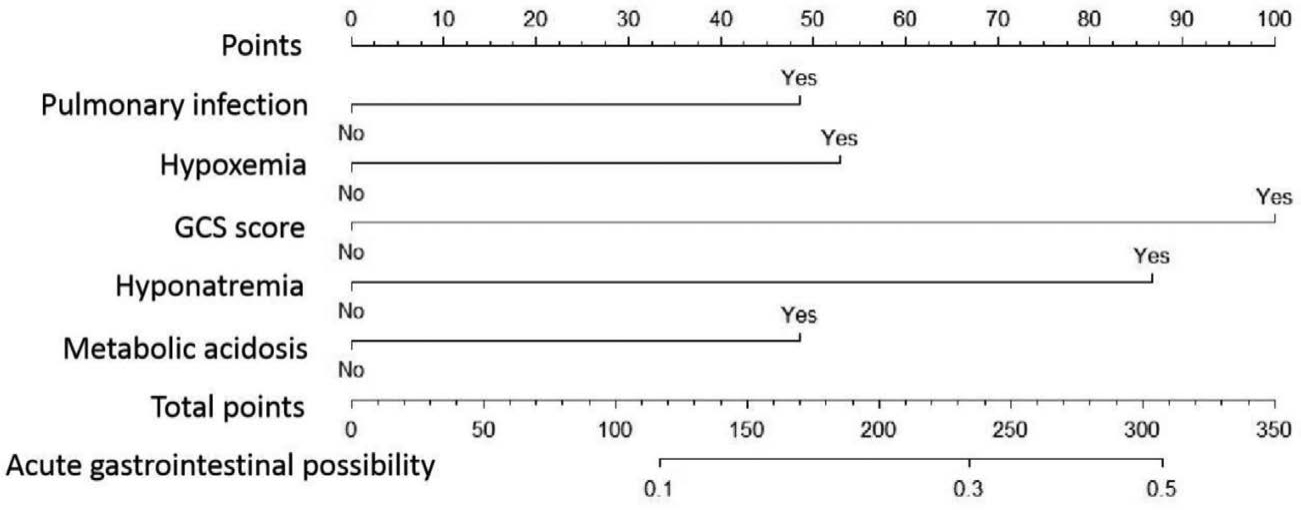
Nomogram of AGF in patients with severe traumatic brain injury. AGF = acute gastrointestinal failure.

The risk factors of AGF in patients with severe traumatic brain injury were used as independent variables to construct a prediction nomogram model. The nomogram formula was as follows: logit P = −4.998 + 0.858 × pulmonary infection + 0.923 × hypoxemia + 1.488 × GCS score ≤ 8 points + 1.274 × hyponatremia + 1.020 × metabolic acidosis. The nomogram prediction model was constructed according to the 5 independent predictors selected by multivariate logistic regression analysis, as shown in Figure 1. Each predictive factor in the nomogram has its corresponding score value, which can be added to get the total score. According to the total score on the risk axis, the probability of occurrence of AGF is obtained.

## 12. Validation of the nomogram model

**Figure 2.**
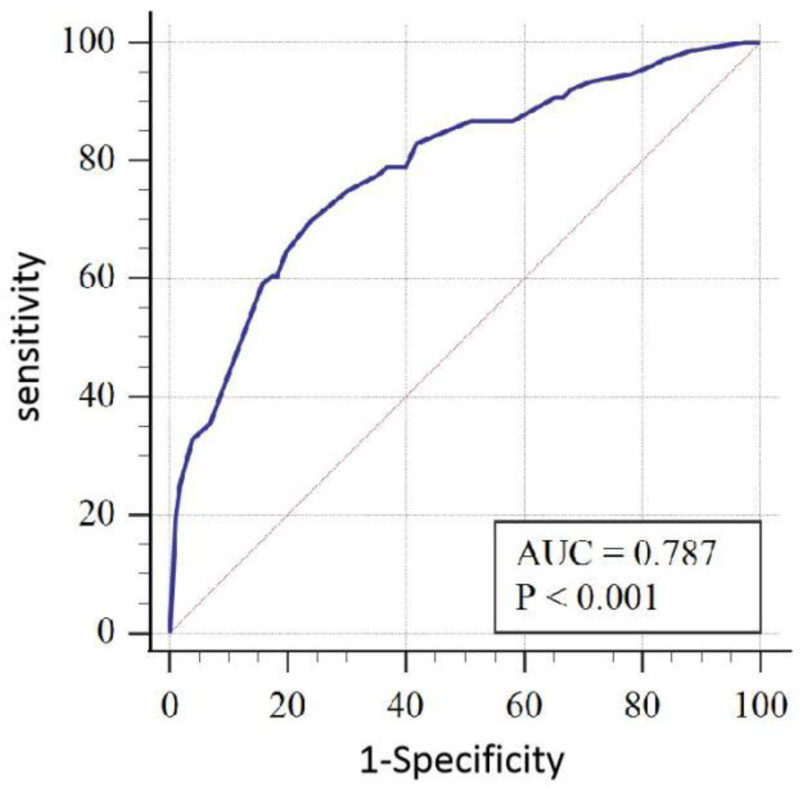
ROC curve of the prediction nomogram for the risk of AGF. AGF = acute gastrointestinal failure, ROC = receiver operating characteristic.

**Figure 3.**
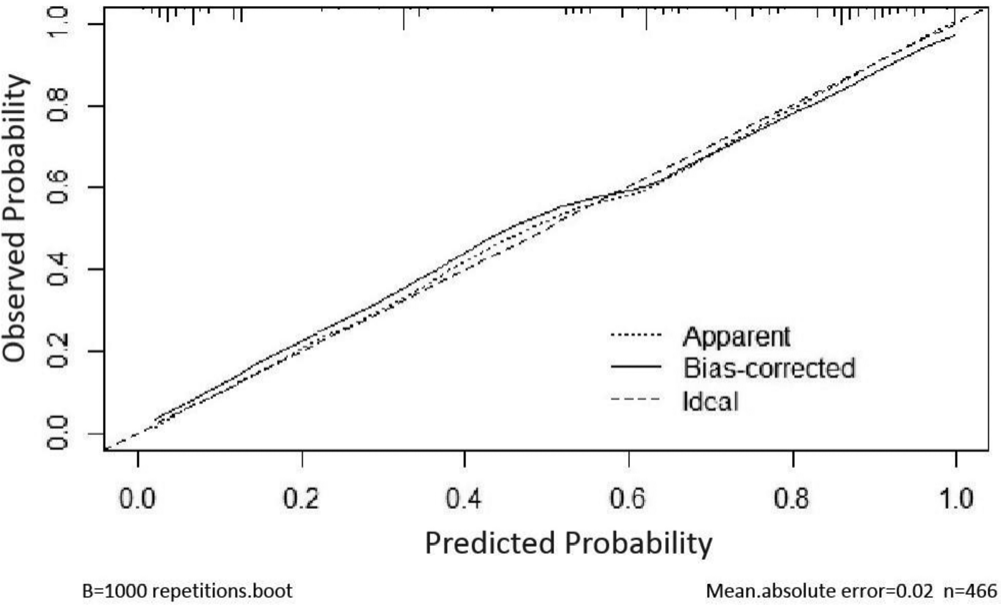
Calibration curve of training group.

**Figure 4.**
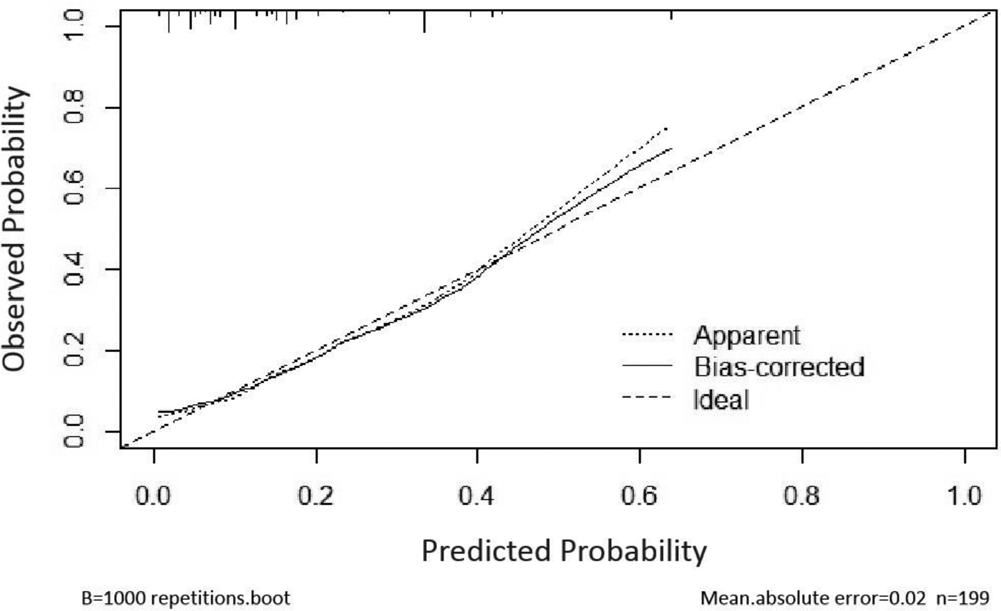
Calibration curve of validation group.

The receiver operating characteristic curve (ROC) was used to evaluate the prediction model, and the area under the ROC curve (AUC) = 0.787, *P* < .001, 95% CI (0.831–0.909), maximum Youden index is 0.4589, where sensitivity is 0.70 and specificity is 0.76, indicating that the prediction model has good diagnostic value, as shown in figure 2. The results of Hosmer–Lemeshow goodness of fit test in this training set showed that χ2 = 4.828, *P* = .681 > 0.05. In addition, the model was verified by the validation group, and the results of Hosmer–Lemeshow goodness of fit test in the validation group showed χ2 = 12.712, *P* = .122 > 0.05, indicating that the nomogram established this time fits well with the real data, that is, the logistic regression results can truly and reliably reflect the relationship between the original variables and the occurrence of AGF in patients. Moreover, the calibration curves in Figures 3 and 4 are close to the actual curves, indicating that the forecast risk predicted by the prediction nomogram model is in good agreement with the actual situation.

## 13. Discussion

### 13.1. Patients with severe traumatic brain injury have a high incidence of acute gastrointestinal failure

The results of this study showed that the incidence of AGF in patients with severe traumatic brain injury was 16.3%, which was consistent with existing literature reports.^[14]^ Current studies suggest that brain-gut regulation involves complex neural, humoral regulation and immune mechanisms, in which the central network system composed of prefrontal cortex, limbic and brainstem regulates the autonomic nervous system, thereby controlling visceral movement and neurosecretion functions. Therefore, the autonomic nervous system plays an important regulatory role in intestinal movement, secretion and immunity.^[15]^ Autonomic dysfunction is common after severe craniocerebral injury, which may be a direct factor affecting the occurrence of gastrointestinal failure.

### 13.2. Analysis of relevant factors in prediction nomogram model of acute gastrointestinal failure in patients with severe traumatic brain injury

2.1 The more severe pulmonary infection and lower blood oxygen saturation in patients with severe traumatic brain injury, the higher the risk of AGF

This study found that pulmonary infection and hypoxemia in patients with severe traumatic brain injury were positively correlated with the risk of AGF, which was consistent with previous research results.^[16]^ Secondary lung infection directly affects the oxygenation of the body, aggravates the oxidative stress response, inflammatory response, immunosuppression and other injuries of the body, further aggravates the original traumatic brain injury, and even leads to sepsis, MODS/MOF, and even death.^[17]^ However, the use of antibiotics may disturb the normal flora in the intestine, lead to the imbalance of the intestinal flora, further aggravate the damage of intestinal structure and function, and thus induce the occurrence of AGF.^[18]^ In addition, when the body is under ischemia and hypoxia stress, the excitability of sympathetic nerve increases, the blood flow decreases and the blood vessels of gastric mucosa spasm contraction. In the state of ischemia and hypoxia, gastrointestinal mucosal energy metabolism disorders, oxygen free radicals and other damage factors increase sharply, damage factors destroy the defense function of gastrointestinal mucosa, and eventually lead to the destruction of gastric mucosal barrier, and gastrointestinal mucosal tissue degeneration and necrosis.^[19]^ It is suggested that nurses should cooperate with doctors in the clinical work to actively control patients’ pulmonary infection, maintain sufficient oxygen supply, and do a good job in gastrointestinal protection.

### 13.3. The lower the GCS score and the lower the serum sodium level of patients with severe traumatic brain injury at admission, the higher the risk of acute gastrointestinal failure

In this study, it was found that GCS score ≤ 8 and hyponatremia were positively correlated with the risk of AGF in patients with severe traumatic brain injury on admission, which was consistent with previous research results.^[16]^ The lower the GCS score, the more serious the traumatic brain injury, the lower the body immunoresistance, the lower the gastric mucosal defense ability, and the gastric mucosal lesions are easy to occur under stress. Meanwhile, the lower the score, the stronger the stress response, the more gastric acid secretion, the more serious the damage of gastrointestinal mucosal defense mechanism, and the higher the incidence of stress gastric mucosal lesions.^[20]^ In addition, hyponatremia can aggravate cerebral edema, further aggravate cranial hypertension and intracranial ischemia and hypoxia. Changes in osmotic pressure caused by hyponatremia may be related to gastrointestinal edema, changes in gastrointestinal membrane potential and other factors, which directly affect the structure and function of gastrointestinal cells and promote the occurrence of AGF after severe traumatic brain injury.^[21]^

### 13.4. The more severe the degree of metabolic acidosis in patients with severe traumatic brain injury, the higher the risk of acute gastrointestinal failure

This study found that metabolic acidosis was positively correlated with the risk of AGF in patients with severe traumatic brain injury. The increased release of H^+^ in acidosis directly damages the gastric mucosa, and the increased release of intracellular lysosomal enzymes destroys the lysosomal membrane, leading to the destruction of gastric mucosal epithelial cells. After craniocerebral injury, anaerobic metabolism is accelerated, lactic acid and hydrogen ions accumulate in the body, which aggravate intracellular and extracellular acidosis, decrease arterial blood pH value, local H^+^ retrograde diffusion of gastric mucosa, reduce gastric mucosal barrier function, damage gastric mucosal cells, rupture and hemorrhage of capillaries, and lead to bleeding from stress ulcer caused by severe traumatic brain injury.^[22,23]^

## 14. The prediction nomogram model of acute gastrointestinal failure in patients with severe traumatic brain injury has good prediction effect

In this study, according to the results of multivariate logistic regression analysis, the corresponding risk prediction nomogram model was established, and the ROC curve was drawn to verify the prediction model. The area under the ROC curve was obtained as AUC = 0.870, *P* > .001, 95%CI (0.831–0.909). The Hosmer–Lemeshow goodness of fit test and calibration curves was carried out on the data by the training group and the verification group, and the result showed that the regression established in this time was well fitted to the real data. All the above results showed that the prediction model was accurate and reliable.

## 15. Conclusion

Based on the risk factors of AGF, this study constructed a prediction nomogram model for the risk of AGF in patients with severe traumatic brain injury. The model has good prediction ability and will provide early warning information for patients with severe traumatic brain injury. Early identification of the risk of AGF will help avoid physical dysfunction, readmissions and death, reduce healthcare costs and improve patient quality of life.

## Contributions

MYD collected the clinical information of patients, performed the statistical analysis, and completed the writing of the manuscript; LF assisted in collecting the patients’ clinical information and writing the manuscript; SLD and HCM helped them; MYD designed the main study and critically revised the manuscript. All authors read and approved the final manuscript.

## Author contributions

**Conceptualization:** Lidi Shen, Chunmin Huang.

**Data curation:** Yadi Mao, Fei Li.

**Formal analysis:** Yadi Mao, Lidi Shen.

**Investigation:** Yadi Mao, Fei Li.

**Methodology:** Yadi Mao.

**Project administration:** Lidi Shen, Chunmin Huang.

**Resources:** Fei Li, Lidi Shen.

**Software:** Fei Li, Lidi Shen.

**Supervision:** Lidi Shen, Chunmin Huang.

**Validation:** Lidi Shen.

**Writing – original draft:** Yadi Mao.

**Writing – review & editing:** Yadi Mao, Chunmin Huang.
